# Do Psychological Factors Influence the Elastic Properties of Soft Tissue in Subjects with Fibromyalgia? A Cross-Sectional Observational Study

**DOI:** 10.3390/biomedicines10123077

**Published:** 2022-11-30

**Authors:** Santiago Navarro-Ledesma, María Aguilar-García, Ana González-Muñoz, Leo Pruimboom, María Encarnación Aguilar-Ferrándiz

**Affiliations:** 1 Department of Physiotherapy, Faculty of Health Sciences, Campus of Melilla, University of Granada, Querol Street 5, 52004 Melilla, Spain; 2 Clinica Ana Gonzalez, Avenida Hernan Nuñez de Toledo 6, 29018 Malaga, Spain; 3 PNI Europe, 2518 JP The Hague, The Netherlands; 4 Instituto de Investigación Biosanitaria ibs. GRANADA, 18012 Granada, Spain; 5 Department of Physiotherapy, Faculty of Health Sciences, University of Granada, Avenida de la Ilustración 60, 18071 Granada, Spain

**Keywords:** fibromyalgia, chronic pain, strain elastography, autonomic nervous system, psychological, kinesiophobia, pain catastrophising, self-efficacy

## Abstract

Nowadays, there is evidence related to the impact that psychological factors have on symptoms, specifically vegetative ones, and on the autonomic nervous system in patients with fibromyalgia (FM). However, there are no studies to correlate the level of association between psychological factors and the elastic properties of tissue in the FM population. Elastic properties of soft tissue reflect age- and disease-related changes in the mechanical functions of soft tissue, and mechanical failure has a profound impact on morbidity and mortality. The study has a cross-sectional observational design with 42 participants recruited from a private clinic and rehabilitation service. The Pain Catastrophizing Scale, Tampa Kinesiophobia Scale and Self-Efficacy Scale were used to assess psychological factors. The elastic properties of the tissue in the characteristic painful points, which patients suffering from FM described, were assessed by strain elastography. A low and significant level of association was found between pain catastrophising scale (PCS) and the non-dominant lateral epicondyle (r = −0.318; *p* = 0.045). Kinesiophobia was found to be related to the dominant lateral epicondyle (r = 0.403; *p* = 0.010), the non-dominant knee (r = −0.34; *p* = 0.027) and the dominant forearm (r = 0.360; *p* = 0.010). Self-Efficacy showed a low level of association with the non-dominant supraspinatus (r = −0.338; *p* = 0.033) and the non-dominant medial epicondyle (r = −0.326; *p* = 0.040). Psychological factors and the elastic properties of tissue seem to be associated in patients suffering from FM. The most profound association between psychological factors and non-dominant parts of the body could be related to neglect and non-use of those parts of the body.

## 1. Introduction

Fibromyalgia (FM) can be defined as a multi-symptom disorder of uncertain origin that manifests with widespread pain, fatigue and sleep problems, anxiety, cognitive disturbances and degenerative or inflammatory behavioural disturbances [1,2,3,4,5]. Additionally, the American College of Rheumatology (ACR) includes specific criteria such as “the presence of generalized pain which lasts for three months, a digital pressure of 4 kg which causes the sensation of pain, and widespread pain with hypersensitivity measured using a map of the characteristic musculoskeletal points to differentiate the diagnosis of fibromyalgia from other rheumatic and painful disorders” [3,4,5,6,7]. Furthermore, according to the 2016 criteria, “a patient must have (1) a WPI of ≥7 and an SSS of >5 OR a WPI of 4–6 and an SSS of ≥9; (2) Widespread pain, defined as pain in at least four of five regions; and (3) symptoms that have been generally present for at least three months. Moreover, there is no exclusion from fibromyalgia criteria for other diagnoses” [7]. 

The prevalence of FM in the general population varies from 0.5% to 5%, and can be up to 15.7% in a clinical setting, with the prevalence in Spain being 4.2% in women and 0.2% in men [6,8]. Although this syndrome can appear in all ages, age causes the prevalence of FM to increase, rising in middle age (50–59 years) and decreasing in the oldest age groups (80+ years) [9]. 

Even though the origin of FM remains unknown, current evidence defines it as a central sensitisation syndrome which involves an alteration in the hypothalamic–pituitary axis (HPA). Hence, ascending and descending pain pathways, as well as neurotransmitters, can be altered, which could be the cause of increased pain responses and the altered circadian variations in blood pressure in patients suffering from FM [10,11].

Furthermore, chronic pain is a condition that includes multiple factors such as central sensitisation, the presence of a low-grade inflammatory state, and alterations in regions of the brain responsible for pain processing and behaviour [12]. The relationship between psychological factors and autonomic symptoms is well known [12,13]. In this regard, previous studies have shown an association between kinesiophobia, catastrophising, disability and vegetative symptoms in people with FM [2], as well as identifying stress as a core element in understanding the different psychological responses that FM patients experience [12,14]. Patients suffering from FM very often show early aging symptoms and disease morbidity [15]. Furthermore, FM is associated with an increase in mortality in patients suffering illnesses such as rheumatoid arthritis and widespread pain in general, although no association was found in patients with cancer (Wolfe 2020). Elastic properties of soft tissue reflect age- and disease-related changes in the mechanical functions of soft tissue, and mechanical failure has a profound impact on morbidity and mortality [16]. Low-grade inflammation, the central entity of chronic disease and FM, also affects elasticity of soft tissue and even cartilaginous tissues [17]. The elastic properties of soft tissue are further influenced or even determined through activity of the sympathetic nervous system [2,18,19,20].

The elastic properties of soft tissue can be measured by ultrasound elastography and loss of elasticity indicates disease progression and/or the effects of non-successful aging [21]. There are no previous studies analysing the relationship between psychological factors and the elastic properties of soft tissue in patients with FM. Defining a possible association of FM with the elastic properties of soft tissue gives the possibility of using elastic property measurement for treatment effectiveness, when and if pain and other symptoms, including psychosocial factors, are associated with those properties.

Whilst several studies on chronic pain and elastic properties have been published [22], there are no studies associating psychological factors and the elastic properties of soft tissue in people with FM.

## 2. Method

### 2.1. Study Design

The present study was a cross-sectional observational design. 

A sample, comprising 42 participants, was recruited from a private healthcare centre in Málaga, Spain. Participants were informed through trial information sheets and formal meetings.

Ethics permission was obtained from the Ethics Committee of Human Research at the University of Granada, Spain, (1044/CEIH/2020) and conducted in accordance with the Declaration of Helsinki. This study is reported in line with the Statement of Recommendations for Intervention Trials (STROBE).

### 2.2. Sample

A sample comprising 42 women with FM participated in the study. All participants were recruited from a private physiotherapy centre in Málaga, Spain, by a physiotherapist. 

The physiotherapist was in charge of providing information about the study to the participants and examining which volunteers met the inclusion and exclusion criteria. 

Two participants were excluded from the study due to some exclusion criteria not being met.

#### 2.2.1. Inclusion Criteria

Aged between 18 and 65.

Diagnosed with FM by a rheumatologist following the ACR classification [7].

#### 2.2.2. Exclusion Criteria

Suffering from any inflammatory, neurological or orthopaedic disease that may impaired balance, hearing, or vision or cognitive impairment that may affect the ability to answer questions.

### 2.3. Outcome Measures

Psychological factors

Psychological factors were measured using the Pain Catastrophizing Scale, the Tampa Kinesiophobia Scale (TSK-11) and the Self-Efficacy Scale. 

Pain Catastrophizing Scale

This validated questionnaire consists of 13 items divided into 3 subsections and aims to measure how catastrophism affects the experience of pain. The first subsection, which includes questions 1 to 5, is called “helplessness” and assesses how the person believes he or she was able to influence their pain [6]. Questions 6, 7 and 13 form the second subsection, called “magnification”, which measures the exaggeration of the threatening properties of pain. Questions 8 to 11 form the last subsection, namely “rumination”, which tests when the patient is not able to stop thinking about their pain [6]. Each section can be evaluated from 1 to 5 with a final score from 0 to 52. High scores will be associated with higher levels of catastrophism.

Tampa Kinesiophobia Scale (TSK-11)

This questionnaire, adapted and validated to the Spanish language, was used to measure fear of movement in the participants in this study since it was proved to be a suitable, reliable and consistent tool to use in people with FM [23]. A total of 11 items comprises the questionnaire. Each item has 4 options ranging from 1 to 4, where 1 corresponds to “strongly disagree” and 4 to “strongly agree”. The total score varies between 11 and 44. High scores imply a greater fear of movement, that is, greater kinesiophobia [24].

Self-Efficacy Scale

The Self-Efficacy Scale was used in this study to measure the perception participants have about their competence to handle a stressful situation efficiently [25]. There is a total of 10 items with a four-point response scale, resulting in a total score of 0 to 44. A higher score is understood to indicate a greater perception of competence to efficiently handle a stressful situation [2,6,26].

Elastic properties of soft tissue

**Figure 1:**
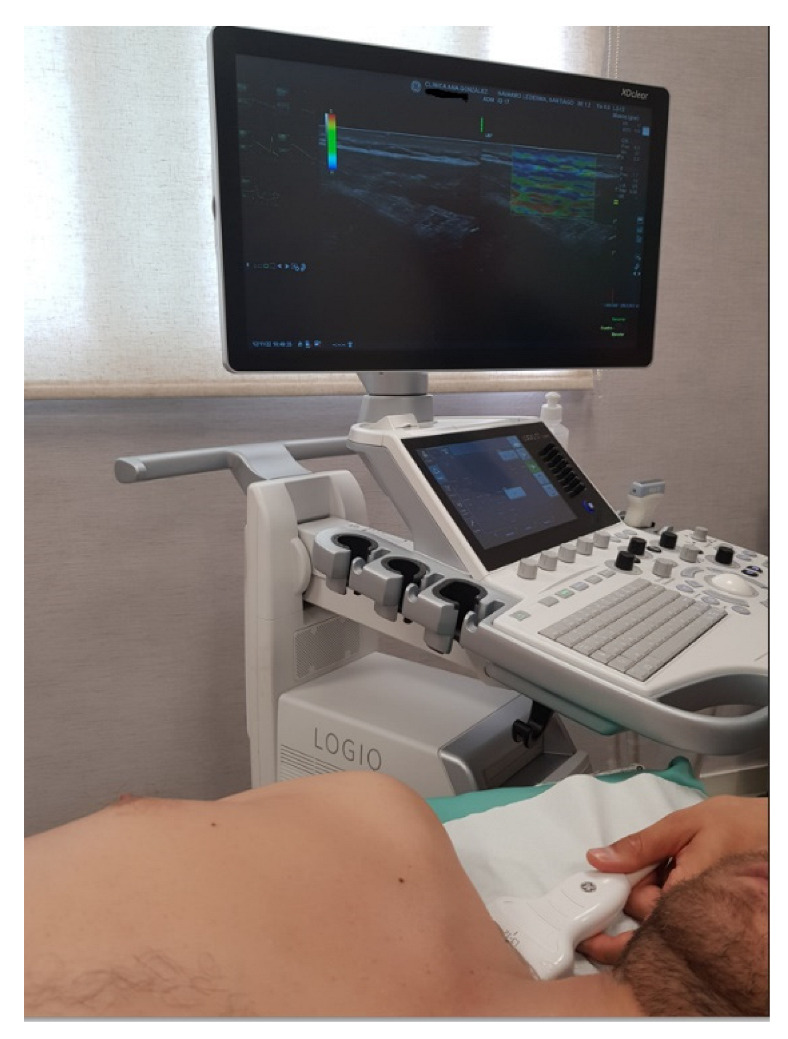
Strain elastography assessment of the midpoint of the upper trapezius.

The elasticity of tissue was assessed by ultrasound elastography (SEL). All measurements were taken by a physiotherapist with 11 years’ experience in ultrasound imaging, using the Logiq S7 with a 15 MHz linear probe (GE Healthcare, Milwaukee, WI, USA). The measurements were obtained by taking images with the transducer placed longitudinally on the muscle fibres, with the centre of the probe positioned on the Myofascial Trigger Points (MTrPs) according to the control point locations [27], and with the patient positioned as indicated in the MTrPs identification protocol [28,29]. Once the transducer was placed at the appropriate point, a tissue compression of 2–5 mm was performed; this was measured by software (see Figure 1). Thirteen points were measured on each side (dominant and non-dominant) according to the ACR criteria [7]: (i) the suboccipital muscle insertions; (ii) the anterior aspects of the intertransverse space at low cervical C5–C7; (iii) the midpoint of the trapezius (upper border); (iv) the supraspinatus origins, above the scapula spine near the medial border; (v) paraspinous, at the level of the mid-scapula, 3 cm lateral to the midline; (vi) the second costochondral junctions (second rib); (vii) the level of the fourth rib at the anterior axillary line (lateral pectoral); (viii) 2 cm distal to the epicondyles; (ix) medial epicondyle; (x) the distal dorsal third of the forearm; (xi) the upper outer quadrants of buttocks in the anterior fold of the gluteal muscle; (xii) the greater trochanter just posterior to the trochanteric prominence; and (xiii) the medial knee fat pad, proximal to the joint line.

In line with the manufacturer’s instructions, and in accordance with previous studies, a 5 mm circular region of the area was selected to measure the exact value of tissue deformation. The resulting values refer to the deformity of the tissue and range from 0 to 6, with 0 being the softest and 6 being the hardest. The value obtained at each point is the average of 3 measurements which results in a more reliable value. Furthermore, only high-quality images were used.

### 2.4. Statistical Analysis

The statistical analysis was performed using the SPSS program, version 23.0 (IBM, Chicago, IL, USA) for Windows. The W of the Shapiro–Wilk test was used to evaluate the normality of the sample. The level of association between the variables (PCS, SE, TSK-11 and SEL in the characteristic painful points in patients with FM) was calculated using the Pearson correlation coefficient for those that followed a normal distribution; for those that did not follow a normal distribution, the Spearman correlation coefficient was used. The results were considered statistically significant when the *p*-value was <0.05 [22]. A correlation displaying values between 0.3 and 0.5 was defined as weak; between 0.5 and 0.7 as moderate; and as strong if the correlation coefficient was more than 0.7. A *p*-value <0.05 was considered statistically significant [30].

## 3. Results

**Figure 2:**
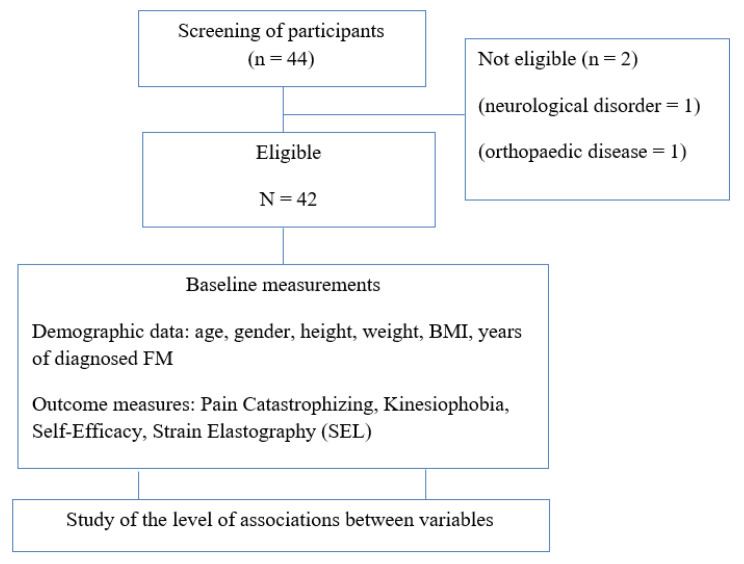
Flow diagram of participants.

The sociodemographic characteristics of the sample are shown in Table 1. A total of 42 women suffering from FM participated in this study. A flow diagram has been included (Figure 2).

### Level of Association between Psychological Factors and SEL in Painful Points

The associations between the Pain Catastrophizing Scale, Tampa Kinesiophobia Scale (TSK-11), Self-Efficacy Scale and strain elastography in the different painful points are presented in Table 2.

A low and significant level of association was found between pain catastrophising (PCS) and the non-dominant lateral epicondyle (r = −0.318; *p* = 0.045). Kinesiophobia was found to be related to the dominant lateral epicondyle (r = 0.403; *p* = 0.010), the non-dominant knee (r = −0.34; *p* = 0.027) and the dominant forearm (r = 0.360; *p* = 0.010). Self-Efficacy showed a low level of association with the non-dominant supraspinatus (r = −0.338; *p* = 0.033) and the non-dominant medial epicondyle (r = −0.326; *p* = 0.040).

The associations between PCS, Self-Efficacy, TSK-11 and the other SEL measurements were not significant.

## 4. Discussion

The aim of the present study was to analyse the relationships between psychological factors (the Pain Catastrophizing Scale, Tampa Kinesiophobia Scale and Self-Efficacy Scale) and the elastic properties of soft tissue in the characteristic painful points that people with FM present.

Our findings show that there is a relationship between psychological factors and the elastic properties of soft tissue, measured by SEL, in patients with FM. We found a low and significant level of association between pain catastrophising (PCS) and the non-dominant lateral epicondyle (r = −0.318; *p* = 0.045). Kinesiophobia was found to be related to the dominant lateral epicondyle (r = 0.403; *p* = 0.010), the non-dominant knee (r = −0.34; *p* = 0.027) and the dominant forearm (r = 0.360; *p* = 0.010). Self-Efficacy showed a low level of association with the non-dominant supraspinatus (r = −0.338; *p* = 0.033) and the non-dominant medial epicondyle (r = −0.326; *p* = 0.040). The repetitive association between psychological factors and the pain points of non-dominant extremities in patients with fibromyalgia can be explained by different mechanisms. Chronic pain can be caused by movement neglect [31], and movement neglect itself is frequently induced by fear [32]. Non-dominant extremities in modern humans are very often neglected and are therefore “forgotten” by the brain, possibly installing fear-induced pain and “freezing” seen in people suffering from frozen shoulder syndrome [33]. The majority of the studied population was right-handed, so the left side has to be considered non-dominant. Our results showed an association between pain points on the left, the non-dominant side, and psychological factors. People with left-sided pain suffer greater pain interference with psychological health than those with right-sided spinal pain [34]. Findings from neuroimaging studies indicate that the right cerebral cortex is preferentially involved in the processing of pain and negative emotions through the convergence of sympathetic afferents in the right anterior insula, which is a major part of the “pain matrix” [35]. The associations between PCS, Self-Efficacy, TSK-11 and the rest of SEL measurements were not significant.

The elasticity and stiffness of soft tissue in fibromyalgia patients have been studied before; however, there is no research studying the relationship between elastic properties of tissue and psychological factors. For this reason, a comparison of our results with other studies is difficult. It is well known that as a consequence of an altered central nervous system, patients with FM exhibit neurovegetative symptoms and both central and peripheral sensitisation, as well as irritable bowel syndrome and the affected gynaecological area, chronic fatigue syndrome, postural orthostatic tachycardia, variations in circadian blood pressure or an excessive response to stimuli such as auditory, cold or mental stress [2,10,11,20,36]. Furthermore, the continuation of pain and symptoms leads to depression and affects mental health [37,38]. In a recent study, an association between vegetative symptoms, pain catastrophising and kinesiophobia has been shown in patients with fibromyalgia [2]. Therefore, we hypothesised that a correlation between psychological factors and elasticity changes in the tissue can be found in people suffering from fibromyalgia. The decreased elastic properties of soft tissue and the correlation with the presence of psychological factors could possibly indicate premature aging effects in people suffering from chronic pain and FM, together with changes in autonomous system activity and low-grade inflammation. These findings are in line with earlier findings in patients suffering from chronic pain and FM [16].

Although not investigated in relation to the elastic properties of soft tissue, other studies already showed a correlation between factors such as pain, vegetative symptoms, disability, kinesiophobia, catastrophism and stress in patients with fibromyalgia. Significantly, Bruna et al. found a correlation between disability, kinesiophobia and psychological factors such as stress in people with FM [13].

To the best of our knowledge, there are no studies analysing the correlation or presence of specific symptoms based on specific psychological factors of interest. In this vein, a unique temporal brain activation of the frontal cortex, and areas of the motor and cingulate cortices during pain anticipation and the subsequent increase in the pain experience, has been seen in patients with FM [39]. Furthermore, social rejection has been shown to alter those areas, which may lead to pain, inflammation and depression, thus activating the immune system as well [39,40]. This shows the inseparable interaction between the psycho-neuro-endocrine-immune systems, and helps to better understand the multifactorial aetiology of FM.

Current studies have measured elasticity and stiffness, in relation to changes in soft tissue properties, using different ultrasound techniques, without finding any significant differences between healthy people and patients with FM [41,42]. However, it is necessary to emphasise that these studies used different ultrasound techniques than those used in our study. In different studies, Muro-Culebras and Cuesta-Vargas used sono-myography and sono-myoelastography to measure trigger-point tissue stiffness, while Karayol and Sibel used Shear Wave Elastography to measure tissue elasticity in rhomboid major muscles. However, in this study, SEL was used, and has been proven to be a useful and reliable technique to measure tissue properties [6,27,28,43,44,45,46,47,48,49]. In addition, SEL measurement was performed, adhering to the proper protocol, on the characteristic Myofascial Trigger Points present in subjects with FM [28].

It is important to highlight that this is the first study analysing the relationship between psychological factors and the elastic properties of the characteristic painful points in patients with FM. Furthermore, all the ultrasound measurements were carried out by an expert with 11 years of experience in this field. On the other hand, some weaknesses must be acknowledged. The study sample is small, only includes women with fibromyalgia and is of a cross-sectional design. In addition, the significance levels of most of the variables were only slightly lower than 0.05; thus, the sample size determination and effect size should become more important in future research. Therefore, the results shown must be interpreted with caution. Additionally, a correlation was only found between psychological factors and the elastic properties; thus, there is no evidence if a causal relationship exists between these three parameters in people suffering from FM.

Future research with a higher number of participants is needed to shed light on these first results which show a relationship between psychological factors and the elasticity of painful points in subjects with FM. These studies should also include follow-up results after successful psychological interventions in patients with FM and the impact of the psychological improvement on elastic properties of soft tissue and pain. Furthermore, it would be of great interest to current research to explore whether specific symptoms and brain areas, including psychological factors, are related. Potential causal relationships between elastic properties and psychological factors could be found through successful intervention studies of both the psychological and tissue factors. These studies would shed light on the impact of the skin–brain axis and its possible bidirectional influence.

## 5. Conclusions

A relationship between pain catastrophising, kinesiophobia, self-efficacy and tissue properties has been found in patients with fibromyalgia. Although only correlated, these results could be used to study the impact of successful psychological interventions on pain and elastic properties of soft tissue in FM patients. The results would clarify a possible causal relationship between psychological factors such as kinesiophobia and elastic properties of soft tissue. More studies are needed to corroborate our findings and shed light on the proposed and as yet unknown underlying mechanisms.

## Figures and Tables

**Table 1:** Sociodemographic Characteristics.

Variable		Women Diagnosed with Fibromyalgia (*n* = 42)		
		Mean ± SD/Frequency (%)	95% CI	W of Shapiro–Wilk (*p*)
Age (years)		52.80 ± 7.90	[50.3, 50.8]	0.95 (0.159)
Weight (kg)		78.20 ± 18.50	[72.3, 84.1]	0.93 (0.019)
Height (m)		1.63 ± 0.04	[1.61, 1.64]	0.96 (0.273)
BMI (kg/m^2^)		29.40 ± 6.36	[27.3, 31.4]	0.87 (<0.001)
Years of diagnosed FM		7.36 ± 1.81	[6.79, 7.92]	0.90 (0.002)
PCS		27.50 ± 12.80	[23.6, 31.5]	0.94 (0.029)
SES		27.40 ± 4.92	[25.8, 28.9]	0.97 (0.600)
TSK-11		27.70 ± 6.94	[25.6, 29.9]	0.95 (0.073)
Occiput	ND	2.58 ± 1.48	[2.11, 3.06]	0.88 (<0.001)
	D	2.40 ± 1.07	[2.05, 2.74]	0.94 (0.101)
Low cervical	ND	2.18 ± 0.97	[1.87, 2.49]	0.96 (0.234)
	D	2.04 ± 0.87	[1.76, 2.32]	0.91 (0.005)
Trapezius	ND	2.38 ± 1.29	[1.96, 2.79]	0.90 (0.003)
	D	2.35 ± 1.28	[1.93, 2.76]	0.90 (0.004)
Supraspinatus	ND	2.04 ± 0.79	[1.93, 2.76]	0.95 (0.128)
	D	2.23 ± 0.88	[1.95, 2.54]	0.96 (0.183)
Paraspinous	ND	2.49 ± 1.16	[2.12, 2.86]	0.96 (0.284)
	D	2.87 ± 1.23	[2.48, 3.26]	0.94 (0.038)
Second rib	ND	2.03 ± 0.86	[1.75, 2.31]	0.95 (0.152)
	D	2.33 ± 0.85	[2.06, 2.61]	0.98 (0.672)
Lateral pectoral	ND	2.31 ± 1.01	[1.99, 2.63]	0.95 (0.47)
	D	1.90 ± 0.78	[1.65, 2.15]	0.93 (0.022)
Lateral epicondyle	ND	1.53 ± 0.85	[1.25, 1.80]	0.83 (<0.001)
	D	1.74 ± 1.03	[1.41, 2.06]	0.92 (0.009)
Medial epicondyle	ND	1.66 ± 1.00	[1.33, 1.98]	0.91 (0.006)
	D	1.57 ± 1.02	[1.24, 1.90]	0.87 (<0.001)
Forearm	ND	2.50 ± 1.22	[2.10, 2.89]	0.93 (0.017)
	D	2.46 ± 1.13	[2.09, 2.82]	0.96 (0.284)
Gluteus	ND	1.60 ± 0.76	[1.35, 1.84]	0.89 (0.001)
	D	1.70 ± 0.68	[1.48, 1.92]	0.94 (0.064)
Greater trochanter	ND	1.89 ± 1.12	[1.53, 2.25]	0.85 (<0.001)
	D	1.99 ± 1.24	[1.60, 2.39]	0.82 (<0.001)
Medial knee fat pad	ND	1.56 ± 1.30	[1.31, 1.82]	0.86 (<0.001)
	D	1.59 ± 1.08	[1.24, 1.93]	0.738 (<0.001)

Note. Data are expressed as mean ± SD for quantitative variables and as frequency (%) for qualitative variables. Abbreviations: BMI (body mass index); CI (confidence interval); PCS (Pain Catastrophizing Scale); SES (self-efficacy scale); TSK-11 (Tampa Kinesiophobia Scale); ND (no dominant); D (dominant); SD (Standard Deviation).

**Table 2:** The association between PCS, TSK-11, Self-Efficacy Scale and strain elastography.

			PCS	SES	TSK-11
Occiput	ND	Rho of Spearman (*p)*	−0.163 (0.314)	−0.155 (0.339)	−0.122 (0.451)
	D	R of Pearson (*p*)	−0.143 (0.377)	−0.289 (0.071)	−0.100 (0.539)
Low cervical	ND	R of Pearson (*p*)	0.216 (0.180)	0.198 (0.220)	0.161 (0.322)
	D	Rho of Spearman (*p*)	−0.175 (0.280)	−0.067 (0.683)	−0.157 (0.333)
Trapezius	ND	Rho of Spearman (*p*)	−0.080 (0.623)	−0.072 (0.660)	0.117 (0.473)
	D	Rho of Spearman (*p*)	0.258 (0.108)	0.028 (0.865)	0.235 (0.145)
Supraspinatus	ND	R of Pearson (*p*)	−0.136 (0.404)	−0.338 * (0.033)	0.024 (0.885)
	D	R of Pearson (*p*)	0.048 (0.769)	−0.181 (0.263)	−0.095 (0.559)
Paraspinous	ND	R of Pearson (*p*)	0.072 (0.658)	−0.143 (0.378)	0.096 (0.554)
	D	Rho of Spearman (*p*)	0.039 (0.813)	−0.066 (0.686)	−0.041 (0.801)
Second rib	ND	R of Pearson (*p*)	−0.053 (0.746)	0.069 (0.673)	0.001 (0.994)
	D	R of Pearson (*p*)	−0.097 (0.553)	−0.070 (0.667)	−0.035 (0.831)
Lateral pectoral	ND	R of Pearson (*p*)	−0.039 (0.812)	0.047 (0.771)	0.235 (0.145)
	D	Rho of Spearman (*p*)	−0.063 (0.701)	0.021 (0.895)	−0.053 (0.744)
Lateral epicondyle	ND	Rho of Spearman (*p*)	−0.318 * (0.045)	−0.112 (0.490)	−0.046 (0.777)
	D	Rho of Spearman (*p*)	0.197 (0.224)	−0.238 (0.140)	0.403 ** (0.010)
Medial epicondyle	ND	Rho of Spearman (*p*)	−0.002 (0.990)	−0.326 * (0.040)	0.161 (0.321)
	D	Rho of Spearman (*p*)	0.143 (0.378)	−0.245 (0.128)	0.304 (0.056)
Forearm	ND	Rho of Spearman (*p*)	0.105 (0.520)	0.212 (0.189)	0.360 * (0.023)
	D	R of Pearson (*p*)	0.194 (0.231)	0.028 (0.864)	0.219 (0.176)
Gluteus	ND	Rho of Spearman (*p*)	−0.074 (0.651)	−0.185 (0.253)	−0.086 (0.598)
	D	R of Pearson (*p*)	−0.084 (0.604)	−0.290 (0.069)	0.033 (0.838)
Greater trochanter	ND	Rho of Spearman (*p*)	−0.147 (0.367)	0.068 (0.678)	−0.304 (0.057)
	D	Rho of Spearman (*p*)	0.032 (0.845)	−0.025 (0.878)	0.043 (0.792)
Medial knee fat pad	ND	R of Pearson (*p*)	−0.186 (0.251)	−0.109 (0.503)	−0.349 * (0.027)
	D	Rho of Spearman (*p*)	−0.079 (0.628)	0.233 (0.148)	−0.082 (0.616)

Note. Abbreviations: PCS (Pain Catastrophizing Scale); SES (self-efficacy scale); TSK-11 (Tampa Kinesiophobia Scale); ND (no dominant); D (dominant); * Significance level: *p* < 0.05; ** Significant level: *p* < 0.01.

## Data Availability

Not applicable.
